# MLN4924, a Protein Neddylation Inhibitor, Suppresses the Growth of Human Chondrosarcoma through Inhibiting Cell Proliferation and Inducing Endoplasmic Reticulum Stress-Related Apoptosis

**DOI:** 10.3390/ijms20010072

**Published:** 2018-12-24

**Authors:** Meng-Huang Wu, Ching-Yu Lee, Tsung-Jen Huang, Kuo-Yuan Huang, Chih-Hsin Tang, Shing-Hwa Liu, Kuan-Lin Kuo, Feng-Che Kuan, Wei-Chou Lin, Chung-Sheng Shi

**Affiliations:** 1 Department of Orthopaedics, School of Medicine, College of Medicine, Taipei Medical University, Taipei 11031, Taiwan; maxwutmu@gmail.com (M.-H.W.); ejaca22@gmail.com (C.-Y.L.); tjdhuang@tmu.edu.tw (T.-J.H.); 2 Department of Orthopedics, Taipei Medical University Hospital, Taipei 11031, Taiwan; 3 Graduate Institute of Clinical Medical Sciences, College of Medicine, Chang Gung University, Taoyuan 33302, Taiwan; 8902029@cgmh.org.tw; 4 Department of Orthopedics, National Cheng Kung University Hospital, Tainan 70101, Taiwan; hkyuan@mail.ncku.edu.tw; 5 Graduate Institute of Basic Medical Science, China Medical University, Taichung 40402, Taiwan; chtang@mail.cmu.edu.tw; 6 Graduate Institute of Toxicology, National Taiwan University College of Medicine, Taipei 10051, Taiwan; shinghwaliu@ntu.edu.tw (S.-H.L.); antibody0123@gmail.com (K.-L.K.); 7 Department of Urology, National Taiwan University Hospital and National Taiwan University College of Medicine, Taipei 11031, Taiwan; 8 Department of Hematology and Oncology, Chang Gung Memorial Hospital, Chiayi 61363, Taiwan; 9 Graduate Institute of Pathology, National Taiwan University College of Medicine, Taipei 10051, Taiwan; weichou8@ms52.hinet.net; 10 Department of Pathology, National Taiwan University Hospital and National Taiwan University College of Medicine, Taipei 11031, Taiwan; 11 Division of Urology, Department of Surgery, Chang Gung Memorial Hospital, Chiayi 61363, Taiwan

**Keywords:** neddylation, NEDD8, chondrosarcoma, MLN4924, endoplasmic reticulum stress, ubiquitin-proteasome system

## Abstract

Chondrosarcoma, a heterogeneous malignant bone tumor, commonly produces cartilage matrix, which generally has no response to conventional therapies. Studies have reported that MLN4924, a NEDD8-activating enzyme inhibitor, achieves antitumor effects against numerous malignancies. In this study, the suppressive effects of MLN4924 on human chondrosarcoma cell lines were investigated using in vitro and in vivo assays, which involved measuring cell viability, cytotoxicity, apoptosis, proliferation, cell cycles, molecule-associated cell cycles, apoptosis, endoplasmic reticulum (ER) stress, and tumor growth in a xenograft mouse model. Our results demonstrated that MLN4924 significantly suppressed cell viability, exhibited cytotoxicity, and stimulated apoptosis through the activation of caspase-3 and caspase-7 in chondrosarcoma cell lines. Furthermore, MLN4924 significantly inhibited cell proliferation by diminishing the phosphorylation of histone H3 to cause G2/M cell cycle arrest. In addition, MLN4924 activated ER stress–related apoptosis by upregulating the phosphorylation of c-Jun N-terminal kinase (JNK), enhancing the expression of GRP78 and CCAAT-enhancer-binding protein homologous protein (CHOP, an inducer of endoplasmic ER stress–related apoptosis) and activating the cleavage of caspase-4. Moreover, MLN4924 considerably inhibited the growth of chondrosarcoma tumors in a xenograft mouse model. Finally, MLN4924-mediated antichondrosarcoma properties can be accompanied by the stimulation of ER stress–related apoptosis, implying that targeting neddylation by MLN4924 is a novel therapeutic strategy for treating chondrosarcoma.

## 1. Introduction

Chondrosarcoma is the third most common primary bone malignancy (after multiple myeloma and osteosarcoma). It is a malignant tumor composed of a hyaline cartilaginous matrix and chondrocytes [1,2,3]. Currently, resection with an adequate margin is the only effective standard treatment for chondrosarcoma. Although less than 10% of patients with grade I chondrosarcomas experience a relapse, the five-year survival of patients with high-grade tumors remains below 60%. Applying conventional chemotherapy regimens to chondrosarcoma generally elicits no therapeutic response; therefore, novel therapies are urgently required for treating patients in advanced disease states, such as those with high-grade, unresectable, metastatic, or refractory cancer [4,5]. Although novel alternative treatments targeting Hedgehog, Src, PI3k–Akt–mTOR, histone deacetylase inhibitors, and angiogenesis have been proposed, clinical evidence of their efficacy is still lacking [6,7]. 

Guaranteeing cellular homeostasis as well as normal cellular function necessitates the maintenance of equilibrium between protein synthesis and degradation [8]. Disordered regulation of the transduction pathway leads to malignancies, drug resistance, metastasis, and progression in cancers [9]. Ubiquitin, a small (8.5 kDa) regulatory protein, is conserved from yeast to mammals, controls protein stability by covalently binding to a target protein, and subsequently tags the protein to the 26S proteasome to induce degradation, which critically controls numerous biological functions in human physiology and pathology [10]. Ubiquitin modification, also known as ubiquitination, is an adenosine triphosphate (ATP)-dependent process performed by three classes of enzymes. The ubiquitin-activating enzyme (E1) activates ubiquitin using the hydrolysis of ATP for an energy source. Subsequently, the activated ubiquitin molecule is delivered to the complex’s second enzyme, known as the ubiquitin-conjugating enzyme (E2). Furthermore, ubiquitin ligase (E3) identifies and links the target substrates with ubiquitin for proteasomal degradation. The E3 ligases are the most influential factors in the ubiquitin–proteasome pathway because of their substrate-specific modulatory functions. Furthermore, cullin-RING E3 ubiquitin ligases (CRLs), the most prominent class of E3, are multisubunit complexes with various substrates that recognize receptors, adaptors, cullin scaffolds, and RING-box proteins [11,12]. Critically, the holoenzyme activity of CRLs is controlled by cullin neddylation, the process of NEDD8 modification, to facilitate a target protein’s ubiquitination. The ubiquitin-like protein NEDD8 is covalently conjugates to the restricted cellular proteins in a manner similar to ubiquitination. The best-characterized neddylation substrates are the cullins, including cullin 1, 2, 3, 4A, 4B, 5, and 7, and PARC in human cells [13]. The E1 NEDD8-activating enzyme (NAE), a heterodimer that consists of regulatory APPBP1 and catalytic UBA3 subunits, activates NEDD8 in an ATP-dependent manner. The activated form of NEDD8 is then transferred to E2 Ubc12 and then is subsequently conjugated to E3, a specific cullin of CRLs, which causes conformational changes in CRLs that facilitate ubiquitin transfer to CRL-targeted proteins for degradation [14]. Some studies have demonstrated that the neddylation pathway is overactivated in various malignancies [15,16], and others have suggested that targeting neddylation to interfere with protein turnover is a promising strategy for treating cancer [17,18]. 

Numerous studies have reported that MLN4924, a selective inhibitor of NAE, is a potential candidate for treating cancers [19,20]. It has been demonstrated to hinder the progression of cancer cells in liver cancer, ovarian cancer, acute myeloid leukemia, urothelial carcinoma, and cervical cancer, both in vitro and in vivo [21,22,23,24,25]. A possible consequence of disrupting the neddylation process is the accumulation of numerous intracellular proteins, which leads to DNA damage, apoptosis, autophagy, and abnormal cellular responses [26]. Nevertheless, the mechanism through which MLN4924 inhibits human chondrosarcoma remains unclear.

In this study, we conducted in vitro and in vivo experiments to explore the mechanism and therapeutic potential of MLN4924 for treating human chondrosarcoma.

## 2. Results

### 2.1. MLN4924 Reduces Cell Viability And Causes Cytotoxicity in Human Chondrosarcoma Cells

**Figure 1:**
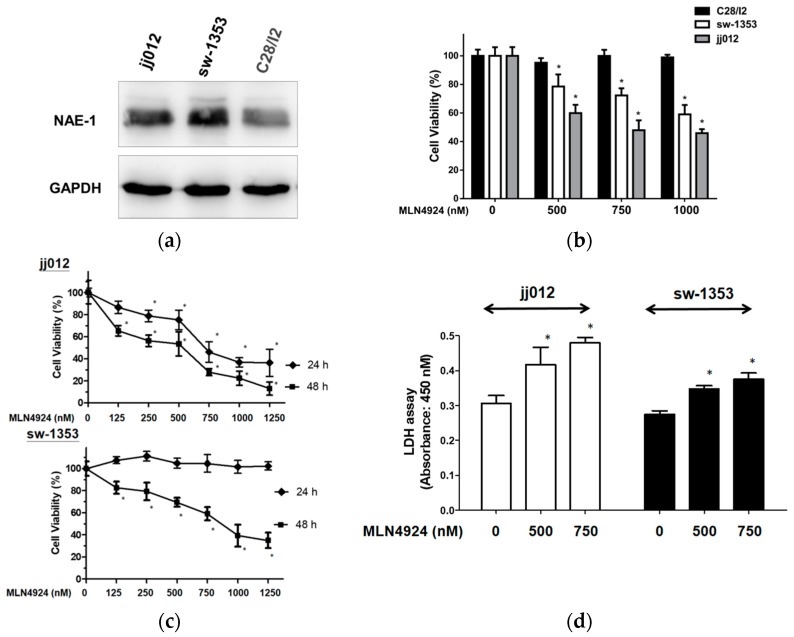
MLN4924 reduced cellular viability and induced cytotoxicity in human chondrosarcoma cells. (**a**) NAE-1 expression in human chondrosarcoma cell lines (jj012, sw-1353), and normal chondrocyte cell line (C28/I2). (**b**) The jj012, sw-1353, and C28/I2 cells were treated with various concentrations of MLN4924 for 24 h. A WST-1 assay was performed to assess cell viability. (**c**) The jj012 and sw-1353 cells were treated with various concentrations of MLN4924 for 24 and 48 h in a WST-1 assay. (**d**) The jj012 and sw-1353 cells were treated with dimethyl sulfoxide ([DMSO], as a control treatment) or MLN4924 (500 and 750 nM) for 48 h, and cytotoxicity was determined through an LDH assay. * *p* < 0.05.

Because an effective treatment for chondrosarcoma is necessary and MLN4924 has exhibited promise for treating various cancers [27,28], the expressing significance of neddylation pathway and effects of MLN4924 on the viability and cytotoxicity in human chondrosarcoma cell lines were first investigated. The chondrosarcoma cell lines jj012 and sw-1353 exhibited higher NAE-1 expression than the normal chondrocyte cell line, C28/I2 (Figure 1a). Furthermore, MLN4924 significantly reduced the cell viability of both jj012 and sw-1353 but not C28/I2 in a dose-dependent manner (Figure 1b), indicating that the upregulated neddylation pathway is a therapeutic target of human chondrosarcoma. Based on the results of Figure 1a,b, the suppressing effects of MLN4924 were only focused on chondrosarcoma cell lines jj012 and sw-1353. The result further showed that MLN4924 significantly reduced the cell viability of both jj012 and sw-1353 in a time-dependent manner (Figure 1c). A lactate dehydrogenase (LDH) assay was used to examine the effect of MLN4924 on the cellular cytotoxicity and cytolysis of chondrosarcoma cell lines. The results also demonstrated that MLN4924 caused significant cytotoxicity in both jj012 and sw-1353 cells (Figure 1d). Generally, jj012 cells were more sensitive to neddylation inhibition than sw-1353 cells in terms of repressed cell viability and induced cytotoxicity. These results indicated that the inhibition of neddylation by the NAE inhibitor MLN4924 holds therapeutic potential for treating human chondrosarcoma.

### 2.2. MLN4924 Suppresses Cell Proliferation by Hindering G2/M Cell Cycle Progression

**Figure 2:**
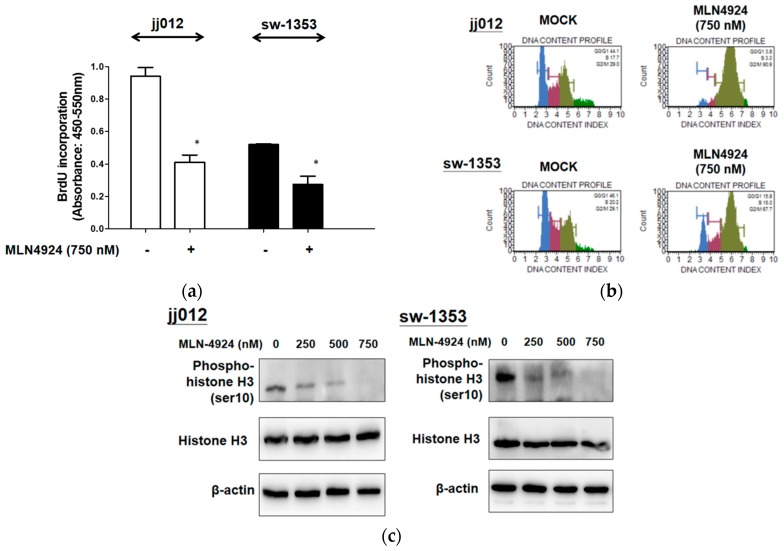
MLN4924 inhibited cell proliferation and caused G2/M cell cycle arrest in two human chondrosarcoma cells. (**a**) The jj012 and sw-1353 cells were exposed to mock (untreated) treatment or MLN4924 treatment (750 nM) for 48 h. After incubation, the status of DNA synthesis in terms of representing cell proliferation was determined using a BrdU incorporation assay. (**b**) Starved jj012 and sw-1353 cells were treated with or without various concentrations of MLN4924 for 24 h. After treatment, cells were subjected to propidium iodide (PI) staining to determine DNA content. (**c**) jj012 and sw-1353 cells were treated with or without various concentrations of MLN4924 (250, 500, and 750 nM) for 48 h. After treatment, the expression levels of cell cycle regulatory proteins, including histone-H3 and phospho-histone-H3 (Ser10), in total cell lysates were analyzed using Western blot analysis. The results are representative of at least three independent experiments. * *p* < 0.05.

We examined the effect of MLN4924 exposure on the proliferation and cell cycle progression of chondrosarcoma cell lines. Figure 2a illustrates that MLN4924 (750 nM) significantly reduced cell proliferation by decreasing BrdU incorporation in jj012 and sw-1353 cells. Moreover, Figure 2b indicates that MLN4924 caused G2/M phase arrest. Figure 2c further illustrates that MLN4924 reduced the phosphorylation of the mitosis marker histone H3 serine 10 in jj012 and sw-1353 cells. These results indicate that MLN4924 suppressed the proliferation of chondrosarcoma cells through inducement of G2/M phase arrest by diminishing histone H3 serine 10 phosphorylation.

### 2.3. MLN4924 Induces Cellular Apoptosis through Intrinsic and Extrinsic Apoptotic Pathways in Human Chondrosarcoma Cells

**Figure 3:**
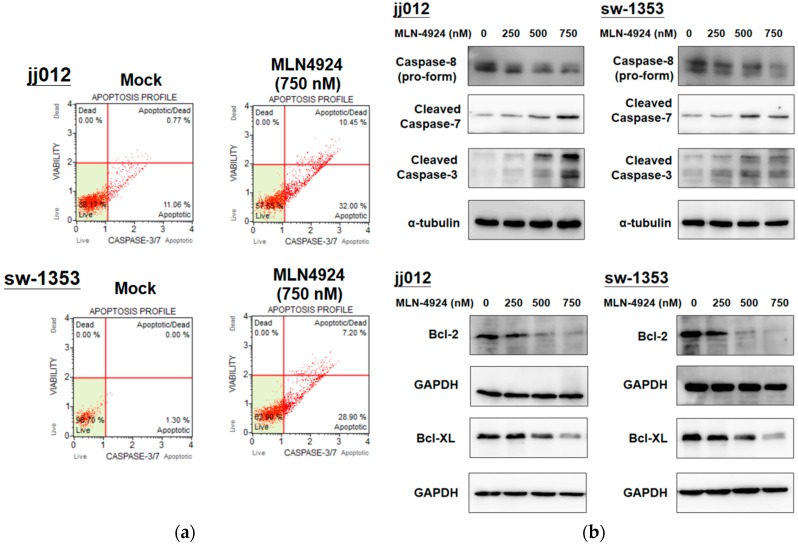
MLN4924 induced apoptosis through caspase-3/7 activation in human chondrosarcoma cell lines. (**a**) The jj012 and sw-1353 cells were treated with 750 nM MLN4924 and DMSO (for the nontreated control group) for 48 h. The activation of caspase-3/7 on apoptotic cells was analyzed using fluorescence-activated cell-sorting flow cytometry. (**b**) After they were harvested, total cell lysates were analyzed by conducting a Western blot analysis that used specifically cleaved caspase-3/-7, casepase-8 (pro-form), Bcl-2, and Bcl-XL antibodies. Similar results were obtained in at least three independent experiments.

After demonstrating that MLN4924 significantly inhibits cell proliferation in chondrosarcoma cells, we examined whether MLN4924 induces apoptosis in chondrosarcoma cells. Furthermore, because activated caspase-3 and -7 are indicators of early-stage apoptosis [29], the effect of MLN4924 on the activation of caspase-3 and -7 in cells was assessed. Flow cytometry analysis was used to examine caspase-3 and -7 activation, and it was determined that MLN4924 (750 nM) treatment considerably stimulated apoptotic caspase-3 and -7 activation in jj012 and sw-1353 cells after 48 h (Figure 3a). Moreover, the expression levels of antiapoptotic Bcl-2 and Bcl-XL, which are intrinsic apoptotic pathway regulators, were dose-dependently downregulated, and the cleavages of caspase-3 and -7 were dose-dependently enhanced by MLN4924 treatment in jj012 and sw-1353 cells (Figure 3b). The protein level of the pro-form of extrinsic caspase-8 was also dose-dependently reduced, indicating an increase in cleaved caspase-8 during apoptosis (Figure 3b). These results (Figure 3) indicated that both intrinsic and extrinsic apoptotic pathways were involved in MLN4924-mediated apoptosis in chondrosarcoma cells.

### 2.4. MLN4924 Promotes ER Stress-Related Signaling and Apoptosis in Human Chondrosarcoma Cells

**Figure 4:**
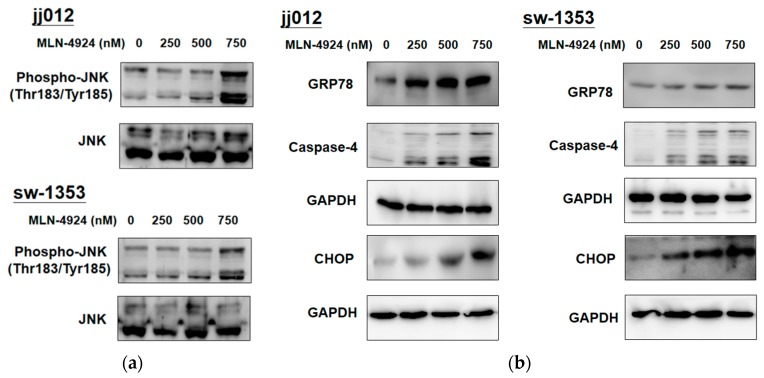
MLN4924 activated ER stress-related apoptosis in human chondrosarcoma cells. The jj012 and sw-1353 cells were treated with various concentrations of MLN4924 (250, 500, and 750 nM) and DMSO (for the control group) for 48 h. After they were harvested, cell lysates were analyzed by a Western blot analysis that used specific antibodies to molecules related to ER stress-induced apoptosis, including (**a**) c-Jun N-terminal kinase (JNK) and phospho-JNK, (**b**) GRP78, CHOP, and caspase-4. The results are representative of at least three independent experiments.

The ER is the cellular organelle responsible for the synthesis of proteins. Once unfolded or misfolded proteins are accumulated in the lumen of the ER, cells activate the unfolded protein response (UPR) to protect themselves against ER stress, which activates ER transmembrane proteins to increase the expression of GRP78 to reestablish ER homeostasis. When ER stress is excessive, the UPR provokes the activation of c-Jun N-terminal kinase (JNK) and expression of the CCAAT/enhancer binding protein-homologous protein (CHOP), which can activate the cleavage of caspase-4 to elicit ER-stress-mediated apoptotic cell death to remove severely damaged cells. In this study, MLN4924-mediated neddylation inhibition interfered with the degradation and turnover of proteins for altering the proteomic profile of chondrosarcoma cells, which a study has determined may augment ER stress-related signaling and induce cell death [30]. Figure 4 illustrates how MLN4924 dose-dependently increased the expression of GRP78 and CHOP, the phosphorylation of JNK, and the cleavage of caspase-4 in jj012 and sw-1353 cells. This finding indicated that MLN4924 increased cellular stress to induce ER stress-associated apoptosis in human chondrosarcoma cells.

### 2.5. MLN4924 Significantly Inhibits Chondrosarcoma Xenograft Growth in Vivo

**Figure 5:**
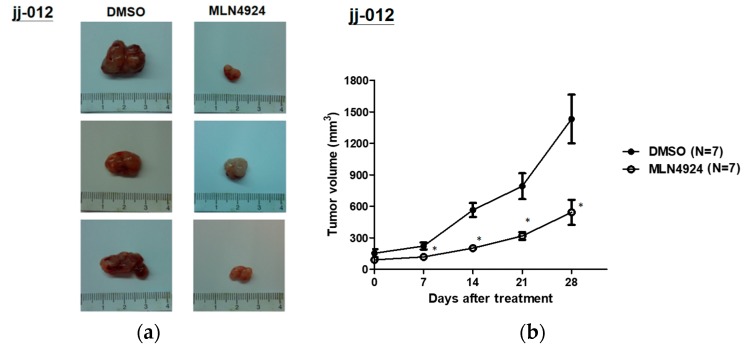

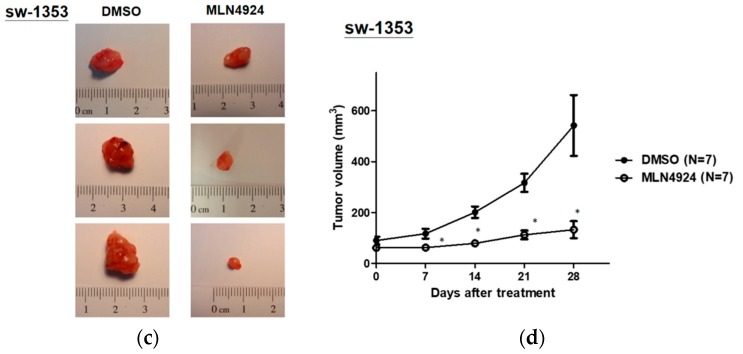
MLN4924 significantly inhibited the growth of the chondrosarcoma xenograft in vivo. Mice with jj012 or sw-1353 cells were grouped for treatment with DMSO or MLN4924 (10 mg/kg/day) through intraperitoneal injection for 5 weeks. (**a**,**c**) Representative images of excised chondrosarcoma tumors from each group of cells. (**b**,**d**) Tumor volumes were recorded to assess tumor growth. On the last day of treatment, tumors from MLN4924-treated and DMSO control mice were compared. * *p* < 0.05 versus DMSO control mice. The weights and volumes of tumors are represented herein as mean ± standard error of the mean; * *p* < 0.05.

Because neddylation inhibition significantly affects the cellular functions of chondrosarcoma cells, the therapeutic efficacy of MLN4924 for suppressing chondrosarcoma xenograft growth in nude mice was examined. Images of jj012 and sw-1353 xenograft tumors are depicted in Figure 5a,c. MLN4924 markedly suppressed the volume of jj012- and sw-1353-xenograft tumors after 4 weeks of treatment compared with the DMSO control group (Figure 5b,d). By contrast, chondrosarcoma cells in the control group exhibited a trend of continual growth, reaching a peak of approximately 750 mm^3^ (Figure 5b,d). Figure 5 indicates that the inhibition of neddylation could inhibit chondrosarcoma cell growth in vivo and suggests that MLN4924 has considerable therapeutic potential for treating human chondrosarcoma.

## 3. Discussion

Chondrosarcoma exhibits a diverse histopathology and clinical behavior; its clinical management is exceptionally challenging because of its inherent resistance to conventional chemotherapy and radiation therapy; thus, new therapeutic approaches are urgently required [31]. Chemotherapy is generally ineffective for treating chondrosarcoma, especially the most frequently observed conventional type and the rare (low-grade) clear-cell variant [32]. Chondrosarcomas with a high percentage of small cells and limited cartilage content are thought to be most sensitive to chemotherapy and radiotherapy, as with other small-cell sarcomas [33]. Moreover, the efficacy of anticancer agents may be impeded by the considerable amount of extracellular matrices and poor vascularity associated with chondrosarcomas, which necessitate the anticancer agents to diffuse over a relatively long distance. This chemoresistance may also be attributed to the expression of the multidrug-resistance 1 gene, *p*-glycoprotein, resulting in resistance to doxorubicin in vitro. Some research has elucidated the molecular events underlying the pathogenesis of this bone malignancy with the goal of developing novel molecularly targeted therapies [34]. Chondrosarcoma signaling involves numerous growth factors and cytokine-regulated signaling pathways such as FGFR1, integrins, ADIPOR, CCR5, and the CXCR4-inducing MAPK-ERK and PI3K-AKT signaling pathways, leading to the activation of MMP, RANKL, VEGF, NF-κB, and p38 MAPK signaling. Chondrosarcoma cells also actively excrete FGF2 and VEGF, which promotes angiogenesis by attracting endothelial cells [27,34]. Furthermore, chondrosarcomas elicit autocrine and paracrine Hh signaling and may be downregulated by the inhibition of Hh pathway signaling by binding to Smoothened [35]. The dependent and independent pathways of EIF2α also regulate the invasion and motility of sw-1353 chondrosarcoma cells and the inactivation of Src, Rac1, and MMP13 by Sal [36]. The dual inhibition of the PI3K/Akt/mTOR and RAF/MEK/ERK signaling pathways has also been demonstrated to have synergistic antiproliferative effects in chondrosarcoma cells [37]. Mutations in *IDH1*/*2*, present in over half of primary conventional chondrosarcomas, render the development of IDH inhibitors a promising treatment option. However, these treatments are still under clinical investigation [38]. Recently, the ubiquitin–proteasome system (UPS) was determined to be associated with chondrosarcoma [39].

Cancer cells take advantage of the UPS by changing the turnover of specific proteins to increase growth and reduce apoptosis. Therefore, the components of the UPS are potential anticancer targets [40]. The UPS is dysregulated in various cancers [41,42]. A study demonstrated that UPS-regulated NF-κB signaling in bone morphogenetic protein-2-activated β1 integrin expression promotes the migration of human chondrosarcoma cells [43]. Moreover, p53, a major tumor-suppressing gene, affects numerous cancers. Low expression of P53 is associated with the metastasis of chondrosarcoma cells [44], indicating the importance of the UPS in chondrosarcoma because the expression level of P53 is controlled by the E3 ubiquitin ligase MDM2, which mediates protein degradation. However, how the UPS is involved in the carcinogenesis and progression of chondrosarcoma is not well characterized. In a recent study, the first-in-class proteasome inhibitor bortezomib inhibited cellular proliferation by stimulating caspase-dependent apoptosis and autophagy [39], suggesting that the UPS is crucial for maintaining the growth of chondrosarcoma cells. However, bortezomib inhibits the overall activity of the UPS, which could cause severe side effects [45]. This indicates that more specific targeting of the UPS is necessary to treat cancers. 

The neddylation pathway of NAE-activated CRLs has been demonstrated to be overexpressed and overactivated in cancer cells [18], indicating that targeting neddylation can more specifically regulate the degradation of proteins and control cancer growth. However, the functional activity and expression pattern of the neddylation pathway in chondrosarcoma cells have not been sufficiently investigated. Researchers have determined that a specific NAE inhibitor, MLN4924, imitates the AMP structure and establishes an adduct with NEDD8 through NAE-1, a protein that participates in the first step of NEDD8 adenylation [46]. MLN4924 can restrict the neddylation of cullins, which leads to tumor-suppressive CRL substrate accumulation. As a result, cell death occurs, leading to the suppression of tumor growth, migration, and invasion. Additionally, other studies have suggested that NEDD8 abrogation may boost carcinogenesis and drug resistance, which has encouraging implications for the development of NEDD8 pathway-regulation strategies for cancer therapy [47,48]. This study first revealed that MLN4924, by specifically targeting NAE-activated neddylation, plays a vital role in suppressing the growth of chondrosarcoma cells, which indicates that the neddylation pathway is crucial for the growth of chondrosarcoma cells and is thus a potential therapeutic target for treating human chondrosarcoma. 

Studies have demonstrated that MLN4924 is less toxic than bortezomib, an agent that blocks proteasome chymotrypsin-like activity [45,49,50]; the lower toxicity may be achieved by specifically targeting CRLs. MLN4924 is currently the focus of numerous preclinical and clinical trials for cancer treatment, possibly because of its low toxicity as well as its specificity [51]. Cullin-RING E3 ubiquitin ligases control protein ubiquitination and resulting degradation. Moreover, they are integral in stress responses, cell cycle progression, signal transduction, and DNA replication [52]. The existence of these aforementioned therapeutic activities accords with the results of our study, which demonstrated that MLN4924 induces caspase activation, cellular stress, and apoptosis in human chondrosarcoma cells. 

We also determined that MLN4924 treatment is associated with the UPR and other proteins related to cellular stress. We determined that MLN4924 activates JNK, which is the corresponding downstream transcription factor belonging to the MAPK signaling pathway. This finding is supported by a related study that demonstrated the sensitivity of the JNK signaling pathway to stress stimuli and controlling cell death, differentiation, and proliferation [53]. Moreover, the current study determined that MLN4924 caused cell death related to the UPR. Protein aggregation in the cytosol or organelles can be induced by CRL substrate protein accumulation, subsequently prompting a UPR [30,54], which then activates ER-resident-chaperone-encoding gene transcription, thereby easing protein folding. The aforementioned UPR is involved in ameliorating cellular stress and restoring ER homeostasis. We further determined that MLN4924 leads to the expression of CHOP, the downstream effector of apoptosis related to the UPR, and GRP78, the key protein for ER integrity and gatekeeper for the UPR. This indicates that apoptosis related to the UPR can induce MLN4924 cytotoxicity in chondrosarcoma cells. 

## 4. Materials and Methods

### 4.1. Cell Culture

Our cell culture experiments were conducted using human chondrosarcoma cell lines jj012 (kindly provided by Sean P. Scully, University of Miami School of Medicine, Miami, FL, USA), sw-1353 (Bioresource Collection and Research Center, Hsinchu, Taiwan), and normal human chondrocyte cell line C28/I2 (SCC043, Merck KGaA, Darmstadt, Germany). These cell lines were maintained in L-15 medium (jj012) [55], RPMI-1640 medium (sw-1353), or Dulbecco’s modified Eagle’s medium/F12 (C28/I2) supplemented with 10% fetal bovine serum (Hyclone, Pittsburgh, PA, USA) and maintained at 37 °C in a humidified atmosphere of 5% CO_2_.

### 4.2. Reagents and Antibodies

MLN4924 (Active Biochem., Hong Kong, China) was prepared in DMSO (Sigma-Aldrich, St. Louis, MO, USA), for cell treatment. Various protein expression levels were examined using Western blot analysis and antibodies such as NAE-1, CHOP, JNK, phospho-JNK (Thr183/Tyr185), cleaved caspase-3/-7, caspase-4, caspase-8 (pro-form), Bcl-2, Bcl-XL, and histone H3, all of which were purchased from Cell Signaling Technology (Danvers, MA, USA). Furthermore, the antibodies against phospho-histone-H3 (Ser10) and GAPDH were obtained from GeneTex (Irvine, CA, USA), and the antibodies against GRP78, β-actin, and α-tubulin were obtained from Santa Cruz Biotechnology (Santa Cruz, CA, USA).

### 4.3. Measurement of Cell Viability

Cell viability was determined using WST-1 cell proliferation and a cytotoxicity assay kit (Biotools, New Taipei City, Taiwan). First, the cells were cultured in 96-well microplates (5000 cells/well) and incubated at 37 °C for 24 h; subsequently, they were exposed to the WST-1 reagent according to the manufacturer’s instructions. The absorbance at 450 nm was detected using a Thermo Scientific Multiskan FC microplate photometer (Thermo Scientific, Rockford, IL, USA).

### 4.4. Lactate dehydrogenase Activity Assay

To determine cytotoxicity, the level of LDH activity in the supernatant of the cultured cell was detected according to the manufacturer’s instructions (Roche, Indianapolis, IN, USA). The absorbance of each sample was measured using the aforementioned microplate photometer.

### 4.5. Apoptosis Assays in Vitro

Whether and to what extent MLN4924 induced apoptosis in chondrosarcoma cell lines was evaluated. The cultures of chondrosarcoma cell lines were treated with various concentrations of MLN4924 in culture medium for 48 h. After treatment, the cells were washed with ice-cold phosphate-buffered saline (PBS) and used for the assay activation of caspase-3/7 according to the manufacturer’s instructions; this was done by quantifying the bioluminescent signal of Z-DEVD-aminoluciferin (Muse Caspase-3/7 assay kit, Millipore, Burlington, MA, USA) using a flow cytometer (BD Biosciences, Franklin Lakes, NJ, USA) [29].

### 4.6. Cell Proliferation Assay

To determine the inhibitory influence of MLN4924 on the proliferation of chondrosarcoma cells, 5-bromo-2′-deoxyuridine incorporation assays (Roche) were performed. Specifically, we seeded the chondrosarcoma cells in 96-well microplates (4000 cells/well) for 24 h, which were then exposed to MLN4924 for 48 h. After treatment, cells were cultured with 5-bromo-2′-deoxyuridine according to the manufacturer’s instructions. We used a Thermo Scientific Multiskan FC microplate photometer operated at dual wavelengths (450–540 nm) to measure absorbance. 

### 4.7. Cell Cycle Analysis

Propidium iodide (BD Biosciences) staining was used to evaluate the effect of MLN4924 on the cell cycle progression of chondrosarcoma cells. Chondrosarcoma cells were starved in serum-free medium to synchronize cell cycle progression. Following starvation, chondrosarcoma cells were treated with various concentrations of MLN4924 for 24 h. Following incubation, the cells were collected and fixed for staining with PI and then analyzed using flow cytometry. The ratios of cells in G0/1, S, and G2/M were calculated using the ModFit computer program (BD Biosciences).

### 4.8. Western Blot Analysis

For protein phosphorylation and expression analyses, the cells were washed with PBS and lysed using cell lysis buffer (Cell Signaling Technology) on ice for 15 min and were subjected to centrifugation at 14,000 rpm at 4 °C for 15 min. After supernatants were harvested, we conducted a bicinchoninic acid protein assay (Thermo Scientific) to determine protein concentrations. Equal quantities of each sample were resolved using SDS-PAGE and subsequently transferred to a polyvinylidene difluoride membrane (Millipore, Billerica, MA, USA). Thereafter, membranes were blocked using bovine serum albumin (5%) with Tris-buffered saline and Tween (TBST) for a minimum period of 1 h. Subsequently, membranes were incubated overnight with the primary antibodies at 4 °C. The resulting membranes were then washed twice using TBST for 10 min and incubated with specific horseradish peroxidase-conjugated secondary antibodies (Genetex, Irvine, CA, USA) for 1 h at room temperature. Subsequently, the antibody-bound membranes were washed twice with TBST and the TOOLS Ultra ECL-HRP substrate detection reagent (Biotools, New Taipei City, Taiwan) was used to visualize protein bands. 

### 4.9. In Vivo Xenograft Experiments

This study was approved by the Institutional Animal Care and Use Committee of National Taiwan University (IACUC No. 20180156, 27 July 2018). The jj012 or sw-1353 cells (5 × 10^5^) were suspended in 200 μL of serum-free medium, and then an equal volume of Matrigel (BD Biosciences) was added. Subsequently, the cells were subcutaneously injected into the dorsal flanks of eight-week-old nude mice (*n* = 7 in each group; Taiwan National Laboratory Animal Center, Taipei, Taiwan). Once their tumors grew to approximately 150 mm^3^, the mice were grouped and subjected to intraperitoneal injections of 10 mg/kg MLN4924 in normal saline twice a day for 5 weeks. The mice that received the treatment were designated as the MLN4924-treated group, and the mice that received only DMSO in normal saline were referred as the control group. Using calipers, tumor volume was measured twice a week using the following formula: V = LD × (SD)^2^/2, where V, LD, and SD represent tumor volume, longest tumor diameter, and shortest tumor diameter, respectively. 

### 4.10. Statistical Analyses

The GraphPad Prism 5 software program (La Jolla, CA, USA) was used to assess data. Data are expressed as mean ± standard deviation. Statistical significance was analyzed using an unpaired Student’s *t* test, and a *p* value of <0.05 was considered statistically significant.

## 5. Conclusions

This study is the first to demonstrate that specifically targeting NAE-activated neddylation could suppress the growth of chondrosarcoma cells, indicating that the activity of the neddylation pathway is critical for the growth of chondrosarcoma cells. MLN4924 caused the activation of ER-stress-associated proteins involved in cellular apoptosis and cell cycle inhibition in chondrosarcoma cells. This may have been achieved through inhibition of the neddylation of cullins, leading to tumor-suppressive CRL substrate accumulation. These findings provide support for using MLN4924 as a potential treatment for chondrosarcoma.
